# Stereotactic versus whole-brain radiotherapy combined with immunotherapy in driver gene–negative NSCLC with brain metastases: a real-world IPTW analysis

**DOI:** 10.3389/fimmu.2026.1815565

**Published:** 2026-06-22

**Authors:** Erha Munai, Lisi Sun, Amu Jike, Dan Tao, Nan Li, Jiang He, Yu He, Liang Du, Wei Zhou, Yongzhong Wu, Dingyi Yang

**Affiliations:** 1 Department of Radiation Oncology, Chongqing University Cancer Hospital, Chongqing, China; 2 Affiliated Hospital of North Sichuan Medical College, Nanchong, China

**Keywords:** brain metastasis, immunotherapy, inverse probability of treatment weighting, non-small cell lung cancer, stereotactic radiotherapy, whole-brain radiotherapy

## Abstract

**Background:**

Brain metastases (BMs) are a common and prognostically unfavorable complication of non-small cell lung cancer (NSCLC). Although the combination of immune checkpoint inhibitors (ICIs) with radiotherapy has shown potential in the treatment of BMs, there is a lack of comparative studies specifically contrasting whole-brain radiotherapy (WBRT) versus stereotactic radiotherapy (SRT), each combined with immunotherapy. This study aimed to systematically evaluate the efficacy and safety of WBRT plus immunotherapy (WBRT+I) versus SRT plus immunotherapy (SRT+I) in patients with driver-gene-negative NSCLC accompanied by BMs.

**Methods:**

This single-center, real-world retrospective cohort study enrolled patients with driver gene-negative NSCLC and BMs who received their first course of intracranial radiotherapy at Chongqing University Cancer Hospital between January 2018 and June 2024. Inverse Probability of Treatment Weighting (IPTW) was applied to balance baseline variables between the WBRT+I and SRT+I groups. Survival outcomes were evaluated using Kaplan-Meier analysis and Cox regression. The primary endpoints were overall survival (OS) and intracranial progression-free survival (iPFS). Secondary endpoints included the intracranial objective response rate (iORR), intracranial disease control rate (iDCR), and safety profile.

**Results:**

In this real-world cohort of 158 patients with driver gene-negative NSCLC with BMs, SRT+I demonstrated superior survival outcomes compared to WBRT+I. After IPTW adjustment, SRT+I was associated with significantly prolonged median overall survival (29.3 vs. 19.9 months, *P* = 0.034) and intracranial progression-free survival (14.7 vs. 9.4 months, *P* = 0.038). These advantages remained consistent in the subgroup with ≤4 BMs. Immunotherapy administered after radiotherapy yielded superior survival compared to the reverse sequence. The SRT+I group also achieved a higher intracranial objective response rate (78.6% vs. 61.5%) and disease control rate (95.0% vs. 88.5%), with a lower incidence of documented radiation-induced brain injury (3.75% vs. 10.26%) and no grade ≥3 immune-related adverse events.

**Conclusion:**

In driver-gene-negative NSCLC patients with BMs, SRT+I demonstrates superior survival and intracranial disease control compared to WBRT+I, particularly in those with limited BMs. These findings provide high-level evidence for optimizing individualized clinical strategies.

## Introduction

For clinicians managing advanced non-small cell lung cancer (NSCLC), the development of brain metastases (BMs) signifies a critical juncture, necessitating effective local control while preserving neurocognitive function (1, 2). Whole-brain radiotherapy (WBRT) and stereotactic radiosurgery (SRT) are standard local modalities, yet their selection involves a well-recognized trade-off between intracranial coverage and the risk of neurotoxicity (3–5). The integration of immune checkpoint inhibitors (ICIs), now a cornerstone of systemic treatment for driver-gene-negative NSCLC, has added a new layer of complexity and promise to this landscape (6, 7). Preclinical and early clinical evidence suggests that radiotherapy can synergize with ICIs by triggering *in situ* vaccination effects, potentially improving outcomes for patients with BMs (8–12).

However, a pivotal question remains unanswered in routine clinical practice: when combining radiotherapy with ICIs, is one radiotherapy modality superior? The distinct biological and clinical profiles of WBRT and SRT may lead to differential interactions with the immune system. For instance, the focused, high-dose ablation of SBRT might induce a more robust immunogenic cell death in targeted lesions, whereas WBRT’s broader scope could potentially alter the tumor microenvironment across the brain, possibly affecting the efficacy of concomitant immunotherapy(I) (13, 14). Current evidence is insufficient to guide this choice, as existing studies often group these strategies together or lack direct comparison.

This lack of comparative data creates a significant dilemma for oncologists designing treatment plans for individual patients. Therefore, to address this pressing clinical uncertainty, we conducted this retrospective study to directly compare the real-world efficacy and safety of SRT plus I versus WBRT plus I in patients with driver-gene-negative NSCLC and BMs. Our findings aim to provide actionable insights to inform therapeutic decision-making while awaiting prospective validation.

## Materials and methods

### Patient selection

This retrospective cohort study consecutively enrolled patients diagnosed with driver gene-negative NSCLC and BMs who received their first course of intracranial radiotherapy at the Radiation Oncology Center of Chongqing University Cancer Hospital between January 2018 and June 2024. The inclusion criteria were as follows: (1) Histologically or cytologically confirmed NSCLC, with genetic testing confirming the absence of known driver gene mutations (including EGFR, ALK, ROS1, etc.); (2) BMs confirmed by contrast-enhanced cranial magnetic resonance imaging (MRI), with at least one measurable intracranial lesion according to RECIST v1.1 criteria; (3) Treatment with at least one cycle of ICIs administered within 3 months before or after intracranial radiotherapy; (4) The radiotherapy for BMs was the first such course delivered, including either WBRT or SRT; (5) Availability of complete imaging evaluation data before and after radiotherapy; (6) Complete treatment records and follow-up data. Exclusion criteria included: (1) No receipt of any I; (2) Overall survival of less than one month; (3) Presence of leptomeningeal disease (LMD), which was rigorously excluded based on a combination of radiological and cytological evaluations. Routine exclusion was based on contrast-enhanced brain MRI (e.g., presence of classical linear or nodular enhancement in the subarachnoid space, sulci, or cranial nerves). Furthermore, for patients presenting with clinical symptoms highly suspicious of LMD (e.g., multifocal cranial nerve deficits or intractable headache) despite inconclusive initial brain MRIs, negative findings from whole-spine contrast-enhanced MRI and/or cerebrospinal fluid (CSF) cytology were required for inclusion; (4) Concurrent secondary primary malignant tumors in other organ systems; (5) Incomplete imaging data, treatment regimens, or follow-up information that compromised the evaluation of efficacy or safety.

### Data collection

Study data were extracted from the electronic medical record system of Chongqing University Cancer Hospital to ensure consistency in clinical data collection and patient follow-up. The collected variables included: age, sex, Karnofsky Performance Status (KPS), pathology (WHO classification), BMs count, perilesional edema of BMs, status of extracranial metastases, BMs type(Synchronous: Refer to metastases diagnosed at the time of or within 6 months after the primary lung cancer diagnosis; Metachronous: Refer to metastases diagnosed more than 6 months after the primary lung cancer diagnosis.), maximum diameter of the largest BMs (D-max), history of thoracic therapy, neurological symptoms before radiotherapy (NSB), intracranial treatment response, radiation-induced brain injury, SRT/WBRT(group), PD-(L)1 tumor proportion score (TPS), I sequence(Be-RT: Immunotherapy received within 3 months before the first intracranial radiotherapy; Af-RT: Immunotherapy received within 3 months after the first intracranial radiotherapy), and information on immunotherapy administered after the diagnosis of BMs.

### Treatments

All enrolled patients received systemic chemotherapy according to their pathological type and physical condition. Specific regimens primarily included platinum-based doublet chemotherapy or monotherapy with agents such as pemetrexed, nab-paclitaxel, docetaxel, or gemcitabine. Immunotherapy consisted of anti-PD-1/PD-L1 inhibitors administered either as monotherapy or in combination with the aforementioned chemotherapy. Additionally, in accordance with institutional standard of care, all patients routinely received peri-radiotherapy systemic corticosteroids (primarily dexamethasone) to manage or prevent radiation-induced peritumoral edema.

Radiotherapy for BMs included WBRT and SRT. Standard WBRT was delivered at a prescribed dose of 30 Gy in 10 fractions; the higher doses (up to 45–54 Gy in 15–25 fractions) specified in our cohort represented cases that received a simultaneous integrated boost (SIB) or sequential boost to the gross tumor volumes. SRT was administered either as single-fraction stereotactic radiosurgery (SRS, 20–24 Gy) or as fractionated stereotactic radiotherapy (FSRT, 24–32 Gy in 3–8 fractions) for larger lesions, with the specific dose determined based on lesion volume and proximity to critical organs.

According to our institutional protocol, SRT was the preferred and recommended modality for patients with limited brain metastases (≤4 BMs) to maximize local control and preserve neurocognitive function. However, in routine clinical practice, a subset of patients with ≤4 BMs received WBRT. The primary driver for this selection was socioeconomic; during the study period (especially the earlier years), SRT/SRS incurred significantly higher out-of-pocket costs and was not fully covered by regional basic medical insurance. Following thorough communication between doctors and patients regarding the cost-benefit ratio, these patients actively opted for WBRT. Secondary reasons included unfavorable tumor characteristics (e.g., excessively large size, poor demarcation, or critical locations hindering safe SRT dosimetric planning) and clinical urgency (e.g., severe acute neurological symptoms requiring immediate palliation without the scheduling and quality assurance delays associated with SRT setup).

### Patients’ follow-up

Patients were regularly followed up through review of our hospital’s electronic medical record (EMR) system combined with telephone follow-up. Importantly, telephone follow-up was strictly limited to ascertaining the vital status (overall survival) and the exact date of death for patients who were lost to routine clinical visits or had entered end-of-life hospice care. It was never used for clinical or radiological assessment of tumor response. > Instead, intracranial lesions and treatment responses were exclusively assessed using contrast-enhanced brain MRI at 3-month intervals, evaluated by our radiologists, and documented in the EMR system. The date of the objective imaging examination indicating progression of intracranial lesions was recorded as the time of disease progression. The data cut-off date for this study was July 31, 2025.

### Research endpoints

The primary endpoints of this study were overall survival (OS) and intracranial progression-free survival (iPFS). OS was defined as the time from the initial diagnosis of BMs to death from any cause or the last follow-up. Intracranial efficacy was evaluated strictly according to the Response Assessment in Neuro-Oncology Brain Metastases (RANO-BM) criteria. Accordingly, iPFS was defined as the time from the initial diagnosis of BMs to the first occurrence of intracranial tumor progression or death from any cause. Specifically, an intracranial progression event was defined as a ≥20% increase in the sum of the longest diameters of target intracranial lesions, the unequivocal appearance of any new intracranial metastatic lesions, or both. Secondary endpoints included intracranial objective response rate (iORR), intracranial disease control rate (iDCR), and intracranial disease progression rate (iDPR). Treatment response was assessed using the following categories: complete response (CR), partial response (PR), stable disease (SD), and progressive disease (PD).

### Statistical analysis

To mitigate the impact of potential confounding factors on efficacy evaluation, this study employed inverse probability of treatment weighting (IPTW) to statistically adjust for imbalances in baseline characteristics between groups. The validity of this method has been established in numerous clinical retrospective studies (15–17). Based on clinical expertise, the following covariates were selected to construct the weighting model: age, sex, KPS, pathology, BMs count, edema of BMs, extracranial metastases, BMs type, thoracic treatment, D-max, NSB, field of BMs-RT, PD(L)-1(TPS), and the sequence of immunotherapy(I sequence). To control the influence of extreme values, weights were winsorized at the 1st and 99th percentiles. After weighting, the standardized mean difference (SMD) was used to assess the balance of covariates, with SMD < 0.1 considered indicative of ideal balance and 0.1–0.2 deemed acceptable. This weighting scheme was also applied in subsequent subgroup analyses to enhance statistical power.

Survival analysis was performed using the Kaplan-Meier method, with between-group comparisons assessed by the log-rank test. Key predictors were screened from all candidate variables using the LASSO regression model, and the selected variables were incorporated into a Cox proportional hazards model adjusted by IPTW for multivariable analysis. Subgroup analyses were conducted for patients with ≤4 BMs and for those receiving different I sequence. Additionally, landmark analysis and restricted mean survival time were used to further validate survival differences. A *post-hoc* statistical power assessment was performed using the Schoenfeld method based on the observed hazard ratio and event number. All statistical analyses were two-sided, with a significance level set at α = 0.05. The analyses were conducted under the supervision of a certified biostatistician using R software (version 4.4.1).

## Result

### Patient characteristics before and after IPTW

**Figure 1:**
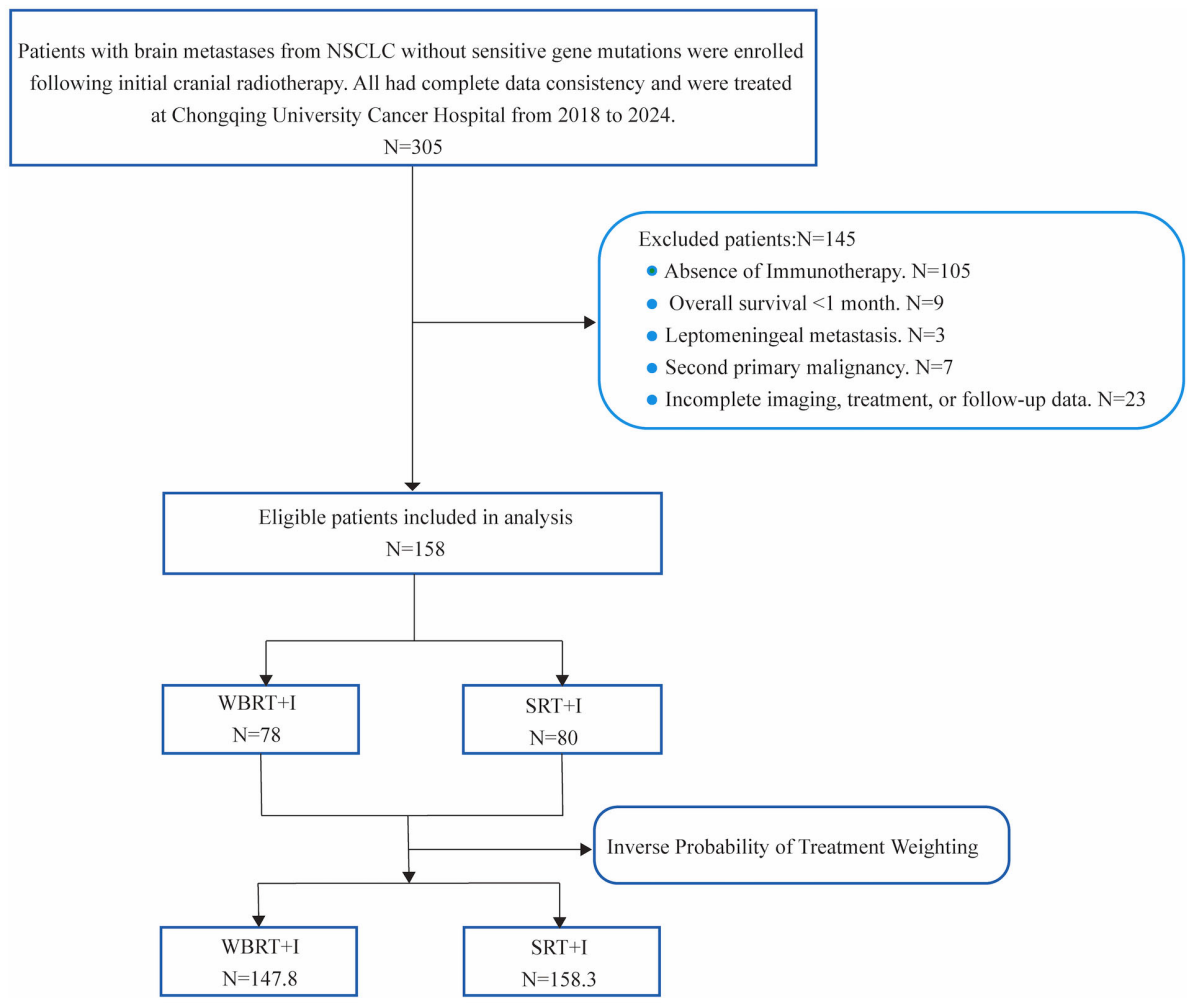
Study flow diagram of patient selection.

This study ultimately included 158 patients with driver gene-negative NSCLC with BMs who received their first course of intracranial radiotherapy at Chongqing University Cancer Hospital (**Figure 1**). Among them, 78 patients were in the WBRT+I group and 80 were in the stereotactic SRT+I group. To minimize baseline confounding bias, IPTW was applied to balance the baseline characteristics (**Table 1**). As of July 31, 2025, the median follow-up for the entire cohort was 32.2 months (95% CI: 27.9–36.5). By the last follow-up, 96 patients (60.8%) had died, and 62 (39.2%) remained alive.

**Table 1:** Patients’ demographics and baseline characteristics before and after IPTW.

Characteristic	Before IPTW		P-value	SMD	After IPTW		P-value	SMD
	WBRT+I, N = 78^1^	SRT+I, N = 80^1^			WBRT+I, N = 147.8^1^	SRT+I, N = 158.3^1^		
Sex			0.125^†^	0.279			0.851^†^	0.047
Male	60 (76.9%)	70 (87.5%)			119.9 (81.1%)	131.3 (82.9%)		
Female	18 (23.1%)	10 (12.5%)			27.9 (18.9%)	27.0 (17.1%)		
Age			0.028^†^	0.384			0.540^†^	0.122
<65	77 (73.1%)	44 (55.0%)			101.4 (68.6%)	99.5 (62.9%)		
≥65	21 (26.9%)	36 (45.0%)			46.4 (31.4%)	58.8 (37.1%)		
KPS			1.000^†^	0.025			0.815^†^	0.046
<80	39 (50.0%)	39 (48.8%)			69.6 (47.1%)	70.9 (44.8%)		
≥80	39 (50.0%)	41 (51.2%)			78.2 (52.9%)	87.4 (55.2%)		
Pathology			1.000^†^	0.014			0.942^†^	0.014
Adenocarcinoma	60 (76.9%)	62 (77.5%)			115.0 (77.8%)	122.3 (77.2%)		
Squamous	18 (23.1%)	18 (22.5%)			32.8 (22.2%)	36.0 (22.8%)		
Extracranial metastases			0.017^†^	0.414			0.417^†^	0.159
No	20 (25.6%)	36 (45.0%)			42.6 (28.8%)	57.3 (36.2%)		
Yes	58 (74.4%)	44 (55.0%)			105.2 (71.2%)	101.0 (63.8%)		
BMs count			<0.001^†^	0.939			0.697^†^	0.088
≤4	40 (51.3%)	72 (90.0%)			101.4 (68.6%)	115.0 (72.6%)		
>4	38 (48.7%)	8 (10.0%)			46.4 (31.4%)	43.3 (27.4%)		
Edema of BMs			0.619^†^	0.110			0.867^†^	0.031
Absent	15 (19.2%)	19 (23.8%)			32.1 (21.7%)	32.3 (20.4%)		
Present	63 (80.8%)	61 (76.2%)			115.7 (78.3%)	126.0 (79.6%)		
BMs type			1.000^†^	<0.001			0.932^†^	0.017
Synchronous	39 (50.0%)	40 (50.0%)			78.2 (52.9%)	82.4 (52.1%)		
Metachronous	39 (50.0%)	40 (50.0%)			69.6 (47.1%)	75.9 (47.9%)		
D-max			1.000^†^	0.005			0.617^†^	0.010
<1.8cm	46 (59.0%)	47 (58.8%)			82.7 (55.9%)	80.6 (50.9%)		
≥1.8cm	32 (41.0%)	33 (41.2%)			65.1 (44.1%)	77.7 (49.1%)		
Thoracic treatment			0.015^‡^	0.474			0.872^‡^	0.095
No	56 (71.8%)	40 (50.0%)			97.8 (66.2%)	98.1 (61.9%)		
Surgery	11 (14.1%)	24 (30.0%)			28.8 (19.4%)	36.3 (23.0%)		
RT	11 (14.1%)	16 (20.0%)			21.2 (14.4%)	23.9 (15.1%)		
NSB			0.152^†^	0.256			0.957^†^	0.011
Absent	43 (55.1%)	54 (67.5%)			93.0 (62.9%)	100.4 (63.4%)		
Present	35 (44.9%)	26 (32.5%)			54.8 (37.1%)	57.9 (36.6%)		
PD(L)-1(TPS)			0.440^‡^	0.205			0.920^‡^	0.075
<1.0%	10 (12.8%)	16 (20.0%)			24.4 (16.5%)	29.7 (18.8%)		
≥1.0%	18 (23.1%)	15 (18.8%)			28.4 (19.2%)	26.8 (17.0%)		
Untested	50 (64.1%)	49 (61.2%)			94.8 (64.2%)	101.8 (64.3%)		
I sequence			0.197^†^	0.233			0.859^†^	0.036
Be-RT	47(60.3%)	57 (71.2%)			92.5 (62.6%)	101.8 (64.3%)		
Af-RT	31 (39.7%)	23 (28.8%)			55.3 (37.4%)	56.5 (35.7%)		

Data are presented as N (%).

WBRT, whole-brain radiotherapy; SRT, stereotactic radiotherapy; I, anti-PD-1/PD-L1 immunotherapy; KPS, Karnofsky Performance Status; BMs, brain metastases; RT, radiotherapy; D-max, maximum diameter of the largest brain metastases; NSB, neurological symptoms before radiotherapy; SMD, standardized mean difference; PD(L)-1(TPS), PD-(L)1 tumor proportion score; Be-RT, Immunotherapy received within 3 months before the first intracranial radiotherapy; Af-RT, Immunotherapy received within 3 months after the first intracranial radiotherapy.

^1^ N (%).

^^†^^ Pearson’s χ² test.

^‡^ Fisher’s exact test.

Before IPTW adjustment, significant differences (defined as *P* < 0.05) were observed in several key clinical characteristics between the two groups. The SRT+I group had a significantly higher proportion of patients aged ≥65 years (45.0% vs. 26.9%, *P* = 0.028) and a significantly higher proportion of patients with ≤4 BMs (90.0% vs. 51.3%, *P* < 0.001) compared to the WBRT+I group. Conversely, the WBRT+I group had a higher proportion of patients with extracranial metastases (74.4% vs. 55.0%, *P* = 0.017). Additionally, differences were observed in the choice of thoracic therapy modalities between the groups (*P* = 0.015).

After applying IPTW, the between-group differences in all baseline characteristics were no longer statistically significant (all *P* > 0.05). Concurrently, the SMD were substantially reduced. Variables with SMD > 0.1 before weighting, such as age, extracranial metastatic status, and particularly the number of BMs (SMD = 0.939), all exhibited post-weighting SMDs below or approaching the 0.1 threshold for balance. This indicates that IPTW successfully balanced all.

### Survival analysis before and after IPTW

**Figure 2:**
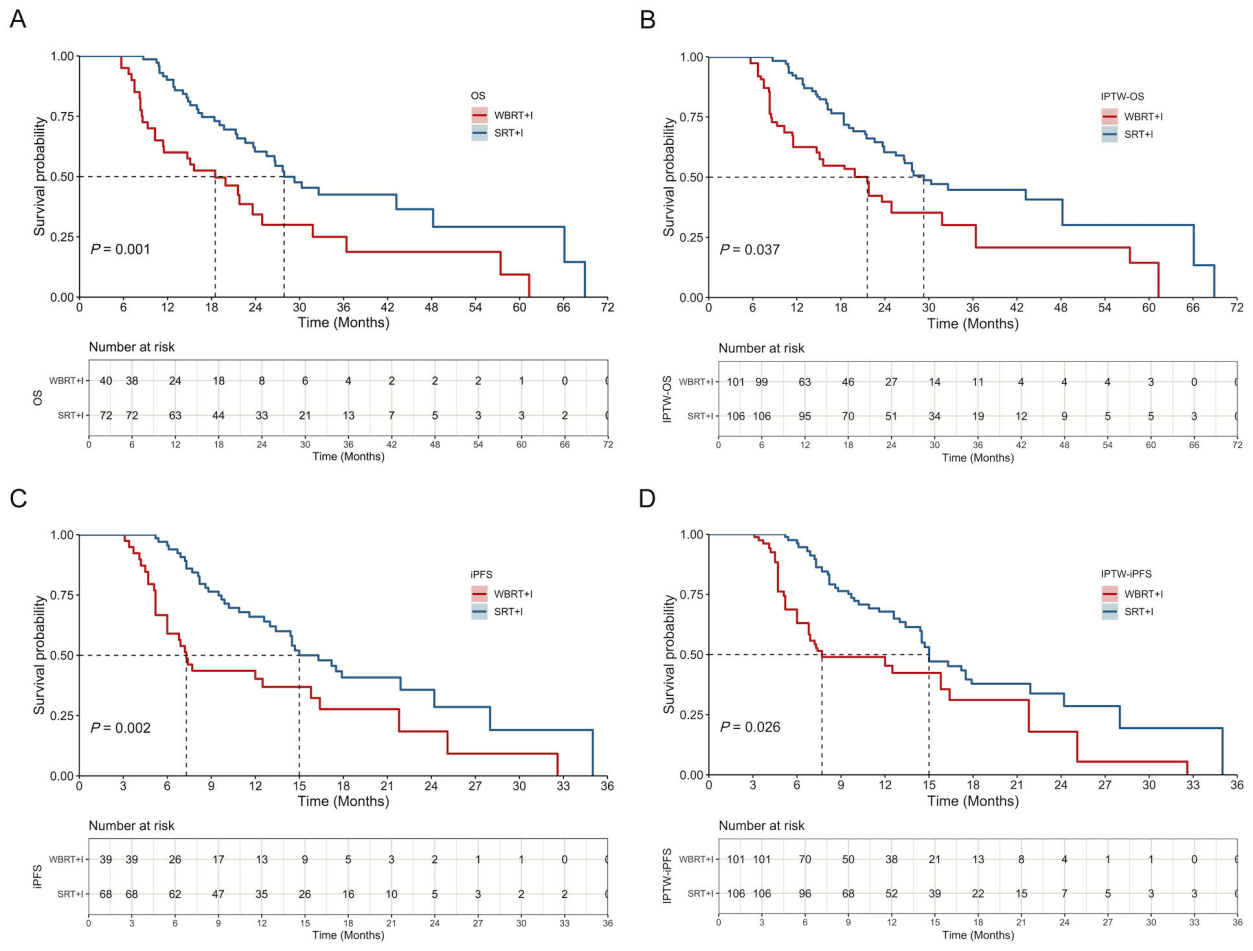
Kaplan–Meier survival analysis according to treatment modality before and after IPTW. **(A)** OS in the overall cohort before IPTW adjustment. **(B)** OS in the overall cohort after IPTW adjustment. **(C)** iPFS in the overall cohort before IPTW adjustment. **(D)** iPFS in the overall cohort after IPTW adjustment.

Prior to applying IPTW adjustment, the median OS from the time of BMs diagnosis for the entire cohort was 22.6 months (95% CI: 18.3-26.9). Intergroup comparisons revealed that the SRT+I group achieved a median OS of 26.7 months (95% CI: 21.2-48.2), which was significantly longer than the 19.7 months (95% CI: 15.1-24.9) observed in the WBRT+I group (*P* = 0.012, **Figure 2A**). Regarding intracranial iPFS, the median iPFS was 14.5 months (95% CI: 12.6-21.9) in the SRT+I group, significantly extended compared to 8.4 months (95% CI: 6.9-15.8) in the WBRT+I group (*P* = 0.004, **Figure 2C**).

After IPTW adjustment, the SRT+I group maintained significant advantages in both OS (**Figure 2B**) and iPFS (**Figure 2D**). Specifically, the adjusted median OS was 29.3 months (95% CI: 23.7-51.2) for the SRT+I group versus 19.9 months (95% CI: 15.1-31.5; *P* = 0.034) for the WBRT+I group. The adjusted median iPFS was 14.7 months (95% CI: 12.6-17.9) for the SRT+I group compared to 9.4 months (95% CI: 6.8-16.4; *P* = 0.038) for the WBRT+I group.

**Figure 3:**
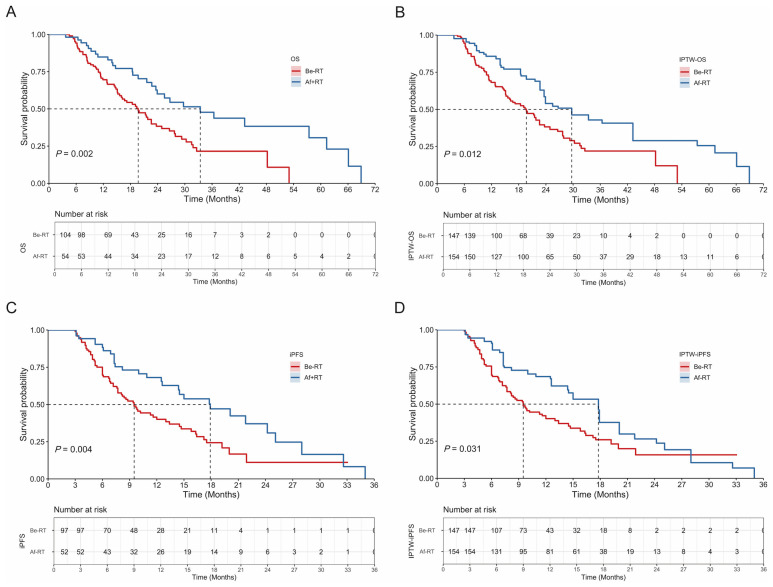
Multivariable Cox regression analysis of prognostic factors for OS and iPFS before and after IPTW adjustment. **(A)** OS before IPTW. **(B)** OS after IPTW. **(C)** iPFS before IPTW. **(D)** iPFS after IPTW.

Key predictors were selected from all candidate variables using the LASSO regression model, and the selected variables were incorporated into an IPTW-adjusted multivariable Cox proportional hazards model. **Supplementary Figure S1** illustrates the relationship between coefficients and log(λ), along with the variable selection process via cross-validation. Before IPTW adjustment, independent prognostic factors for OS included group (P = 0.007), thoracic treatment (*P* = 0.014), and I sequence (*P* < 0.001) (**Figure 3A**); for iPFS, they were group (*P* = 0.004), thoracic treatment (*P* = 0.006), and I sequence (*P* < 0.001) (**Figure 3C**). After IPTW adjustment, the multivariable Cox regression analysis confirmed group (*P* = 0.006) and I sequence (*P* = 0.002) as independent prognostic factors for OS (**Figure 3B**). Significant predictors for iPFS included group (*P* = 0.007) and I sequence (*P* = 0.002) (**Figure 3D**).

### Subgroup analysis

Given the established advantages of SRT in local disease control and neurocognitive preservation, it has become a cornerstone of standard care for patients with oligometastatic BMs (defined as ≤4 BMs). To further investigate the efficacy of SRT combined with I in this specific population, we conducted a focused analysis within this subgroup. Furthermore, the optimal sequencing of I and radiotherapy remains a central and actively investigated question in clinical practice, as it may critically influence the immune response. Therefore, for both subgroup analyses, we applied the same propensity score weighting and survival analysis methods as those used in the primary full-cohort study.

### Cohort with ≤4 BMs

In the subgroup of patients with ≤4 BMs, a total of 112 patients were included, comprising 72 in the SRT+I group and 40 in the WBRT+I group. To control for potential confounding factors, IPTW was also applied to this subgroup to balance baseline characteristics. After weighting, no statistically significant differences were observed between the two groups for any covariates, including sex, age, and patholog (all *P* > 0.05). The standardized mean differences were significantly reduced, indicating that the baseline characteristics achieved an analyzable state of balance after weighting (**Supplementary Table 1**).

**Figure 4:**
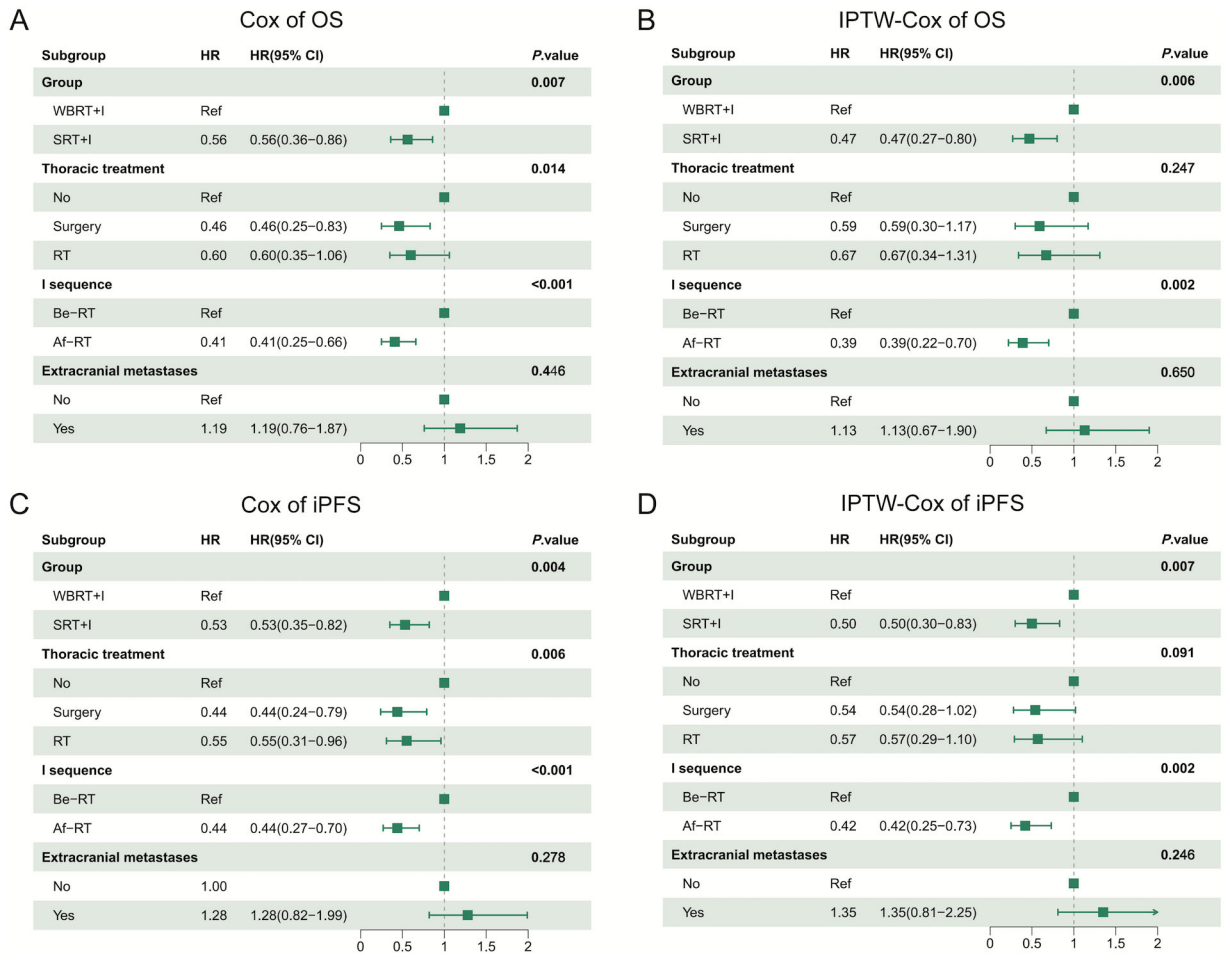
Kaplan–Meier survival analysis in the subgroup with ≤4 BMs before and after IPTW. **(A)** OS before IPTW. **(B)** OS after IPTW. **(C)** iPFS before IPTW. **(D)** iPFS after IPTW.

The median OS for this entire subgroup was 25.5 months (95% CI: 20.6-30.4). In the pre-weighting survival analysis, the SRT+I group exhibited a median OS of 27.9 months (95% CI: 23.9-33.6), significantly superior to the 18.5 months (95% CI: 10.4-26.6) in the WBRT+I group (*P* = 0.001; **Figure 4A**). The median iPFS was 15.0 months (95% CI: 12.2-17.7) and 7.3 months (95% CI: 6.1-8.4) for the SRT+I and WBRT+I groups, respectively (*P* = 0.002; **Figure 4C**). After IPTW adjustment, the SRT+I group maintained its significant advantage: the median OS increased to 29.3 months (95% CI: 25.5-NA), significantly better than the 19.9 months (95% CI: 11.5-31.8) in the WBRT+I group (*P* = 0.037; **Figure 4B**). The median iPFS was 16.3 months (95% CI: 14.4-18.3) for the SRT+I group, significantly longer than the 7.3 months (95% CI: 6.0-16.4) in the control group (*P* = 0.026; **Figure 4D**).

Clinically relevant covariates were selected using LASSO regression (**Supplementary Figure S2**). In the pre-weighting multivariable analysis, independent prognostic factors for OS included group, thoracic treatment, and I sequence; the same factors were identified for iPFS. After IPTW adjustment, the multivariable Cox regression further confirmed that group, thoracic treatment, and I sequence remained independent prognostic factors for both OS and iPFS, retaining statistical significance (**Supplementary Figure 3**).

### I sequence cohort

In the subgroup analysis of the I sequence cohort, 158 patients were included, consisting of 104 in the Be-RT group and 54 in the Af-RT group. The distribution of baseline characteristics for both groups before and after the application of IPTW is shown in **Supplementary Table 2**. After IPTW adjustment, the standardized mean differences for all covariates were below 0.1, indicating good balance in baseline characteristics between the groups.

**Figure 5:**
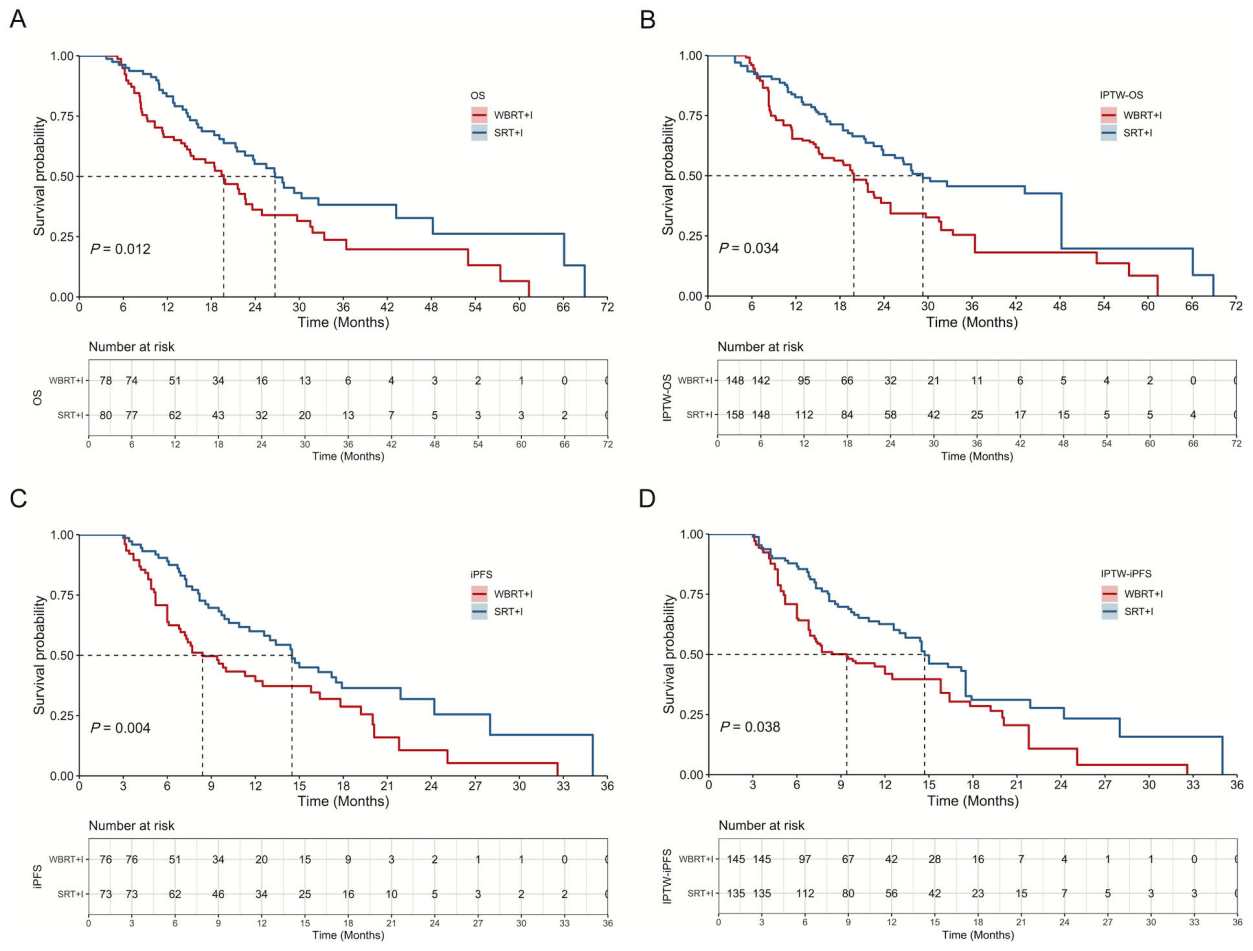
Kaplan–Meier survival analysis by I sequence before and after IPTW. **(A)** OS before IPTW. **(B)** OS after IPTW. **(C)** iPFS before IPTW. **(D)** iPFS after IPTW.

The median follow-up time was 22.6 months (95% CI: 18.4-26.8). In the pre-weighting survival analysis, the Af-RT group demonstrated a significant survival advantage: the median OS was 33.4 months (95% CI: 20.7-46.1), significantly superior to the 19.7 months (95% CI: 15.2-24.2) in the Be-RT group (*P* = 0.002; **Figure 5A**). The iPFS was 17.9 months (95% CI: 12.6-28.0) and 9.5 months (95% CI: 7.7-11.3) for the Af-RT and Be-RT groups, respectively (*P* = 0.004; **Figure 5C**). After IPTW adjustment, the survival advantage of the Af-RT group remained statistically significant. The adjusted median OS was 29.7 months (95% CI: 23.6-NA), significantly better than the 19.7 months (95% CI: 15.6-26.6) in the Be-RT group (*P* = 0.012; **Figure 5B**). The adjusted median iPFS was 17.8 months (95% CI: 12.6-24.2) for the Af-RT group compared to 9.5 months (95% CI: 7.7-13.4) for the Be-RT group (*P* = 0.034; **Figure 5D**).

Clinically relevant covariates were selected using the LASSO regression model (**Supplementary Figure S4**). In the pre-weighting multivariable analysis, independent prognostic factors for OS included I sequence (*P* < 0.001), thoracic treatment (*P* = 0.014), and group(*P* = 0.007) (**Supplementary Figure S5A**); for iPFS, the independent prognostic factors were I sequence (*P* < 0.001), thoracic treatment (*P* = 0.006), and group (*P* = 0.004) (**Supplementary Figure S5C**). The IPTW-adjusted multivariable Cox regression analysis further confirmed that I sequence (P = 0.001), thoracic treatment (*P* = 0.041), and group(*P* = 0.015) were independent prognostic factors for OS (**Supplementary Figure S5B**); significant predictors for iPFS included I sequence (*P* = 0.004), thoracic treatment (*P* = 0.019), and group (*P* = 0.014) (**Supplementary Figure S5D**).

### Efficacy and safety outcomes

Among all 158 patients, the SRT+I group demonstrated superior intracranial efficacy. The iORR was 78.6% (95% CI: 68.2–87.1) in the SRT+I group, higher than 61.5% (95% CI: 49.8–72.3) in the WBRT+I group. The iDCR was 95.0% (95% CI: 87.7–98.6) for SRT+I versus 88.5% (95% CI: 79.2–94.6) for WBRT+I. Furthermore, the iDPR was only 5.0% (95% CI: 1.4–12.3) in the SRT+I group, lower than the 11.5% (95% CI: 5.4–20.8) observed in the WBRT+I group. Detailed efficacy data for each group are provided in **Supplementary Table 3**.

Regarding immune-related adverse events (irAEs), both groups exhibited generally acceptable tolerability profiles (**Table 2**). The incidence of any-grade irAEs was 15.38% in the WBRT+I group and 12.50% in the SRT+I group. The WBRT+I group reported two cases (2.56%) of grade ≥3 irAEs, including one case of acute liver failure, which improved with supportive care, and one case of fatal immune-mediated pneumonitis. No grade ≥3 irAEs occurred in the SRT+I group. The incidence of radiation-induced brain injury was higher in the WBRT+I group (10.26%) compared to the SRT+I group (3.75%), suggesting a more favorable safety profile regarding documented severe cerebral radiotoxicity for SRT+I, although comprehensive neurocognitive outcomes were not assessed.

**Table 2:** Incidence of treatment-related adverse events (TRAEs).

Events, n (%)	WBRT+I (N=78)		SRT+I (N=80)	
	Any grade	Grade> 3	Any grade	Grade> 3
Immune-related TRAEs	12 (15.38%)	2 (2.56%)	10 (12.50%)	
Immune-mediated dermatitis	6 (7.69%)	0	4 (5.00%)	0
Hypothyroidism	3 (3.85%)	0	4 (5.00%)	0
Immune-mediated colitis	1 (1.28%)	0	0	0
Acute liver failure	1 (1.28%)	1 (1.25%)	0	0
Immune-mediated pneumonitis	1 (1.28%)	1 (1.25%)	1 (1.25%)	0
Immune-mediated adrenal insufficiency	0	0	1 (1.25%)	0
Radiation-induced brain injury	8 10.26%)		3 (3.75%)	

## Discussion

This study conducted a real-world cohort analysis that, for the first time, systematically compared the efficacy and safety of whole-brain radiotherapy(WBRT) versus stereotactic radiotherapy(SRT), each combined with immunotherapy (I), in patients with driver gene-negative non-small cell lung cancer (NSCLC) with brain metastases(BMs) receiving their first course of intracranial radiotherapy. Historically, WBRT and SRT have possessed distinct clinical indications, with WBRT primarily serving a palliative role for extensive disease and SRT focusing on definitive local ablation. However, our study addresses a more contemporary clinical dilemma: when a patient is already scheduled to receive systemic immunotherapy, which concurrent intracranial radiation modality yields superior survival and safety outcomes? Directly comparing these two biologically distinct modalities is clinically imperative to optimize combination strategies in the immunotherapy era. Because these two modalities are administered to inherently different patient populations in real-world practice, directly comparing them introduces significant selection bias. Although the study had a retrospective design, we employed Inverse Probability of Treatment Weighting(IPTW) to balance baseline characteristics. All covariates incorporated into the model were selected based on clinical expertise, and balance was assessed using standardized mean differences. Compared with propensity score matching, IPTW achieved sufficient intergroup comparability while preserving a larger sample size. Furthermore, in a study setting with multiple covariates and a moderate sample size, IPTW, through weighted adjustment, avoided the exclusion of unmatched subjects, better reflecting the structure of real-world data and reducing the selection bias potentially introduced by restrictive matching algorithms.

Analyses both before and after IPTW adjustment consistently demonstrated that in the overall population, the SRT+I group was superior to the WBRT+I group in terms of both overall survival(OS) and intracranial progression-free survival(iPFS), suggesting that the choice of radiotherapy technique plays a critical role in patient prognosis in the era of I. Concurrently, the SRT+I group exhibited a higher intracranial disease control rate and lower central nervous system toxicity. In the key subgroup with ≤4 BMs, the survival benefit of SRT+I remained stable both before and after IPTW, offering higher-level real-world evidence for clinical decision-making in this patient population. Further analysis of I timing revealed that initiating I within 3 months after the first intracranial radiotherapy provided significantly superior survival benefit compared to administration within 3 months before radiotherapy, indicating that the treatment sequence may influence clinical outcomes.

Radiotherapy elicits systemic anti-tumor immune responses by inducing immunogenic cell death, which releases a multitude of tumor-associated antigens. This process promotes dendritic cell maturation, activates CD4^+^ and CD8^+^ T cells, and upregulates MHC molecule expression to enhance antigen presentation (18, 19). SRT, as a high-precision radiotherapy modality, can further modulate the local immune landscape. It precisely targets tumors, alters tumor cell antigen expression, stimulates interferon production, and induces immunogenic cell death, thereby fostering a transition of the tumor microenvironment towards a pro-inflammatory state conducive to T cell trafficking from the periphery into tumor sites (14, 20, 21). However, the immune response triggered by radiotherapy alone, including SRT, is often insufficient for achieving durable intracranial disease control, with approximately 30%–50% of patients developing new lesions (22). This limitation stems partly from concurrent activation of negative immunoregulatory mechanisms: firstly, through upregulation of the DNA exonuclease TREX1, which degrades cytosolic damaged DNA and consequently suppresses the critical cGAS–STING immune signaling pathway (23); and secondly, by potentially replacing intratumoral functional T cell clones with peripheral, functionally impaired T cells, thereby sustaining an immunosuppressive microenvironment (24). The application of ICIs effectively counteracts these limitations. Preclinical studies demonstrate a significant synergistic effect when combining radiotherapy with I: radiotherapy-induced release of neoantigens provides specific targets for the immune system, while ICIs, by blocking pathways such as PD-1/PD-L1, reinvigorate CD8^+^ T cells within the tumor microenvironment and reverse T cell exhaustion (13). This combined strategy not only enhances local control in various tumor models but can also elicit the “abscopal effect,” suppressing non-irradiated metastatic lesions and even converting “immunologically cold” tumors into a state sensitive to I (25, 26).

Building upon the I effects of radiotherapy and the synergistic mechanisms with ICIs, accumulating clinical evidence indicates that combining intracranial radiotherapy with I yields superior survival and intracranial disease control in NSCLC patients with BMs. A retrospective analysis by LIAO G et al. (27) of 70 patients demonstrated that combining I with WBRT significantly extended median overall survival compared to WBRT alone (27 months vs. 20 months, *P* = 0.035), without increasing treatment-related adverse events. Another large-scale study involving 315 patients further revealed that combining I with SRS significantly improved overall survival (40 months vs. 8 months, *P* < 0.001) and reduced the 2-year local intracranial failure rate (1.7% vs. 15.7%, *P* = 0.025) compared to SRS alone (28). Recently, the results of the C-Brain trial by Yun Fan et al. (29) in 63 patients (39 patients: SRS; 24 patients: WBRT) showed that brain radiotherapy combined with camrelizumab showed better median iPFS (7.4 months, 95% CI 7.5–15.7) and median OS (20.9 months, 95% CI 13.8–27.7) compared with CAP-BRAIN. Although these studies confirm the synergistic benefits of combining radiotherapy with I comparative research between SRT versus WBRT each combined with I remains limited.

A comprehensive meta-analysis encompassing 46 clinical trials and 3,160 patients with non-small cell lung cancer brain metastases provides crucial supporting evidence: it demonstrated that immunotherapy combined with stereotactic radiosurgery was superior to immunotherapy combined with whole-brain radiotherapy across multiple efficacy endpoints, showing particularly significant advantages in intracranial objective response rate (75% vs. 57%) and intracranial disease control rate (84% vs. 57%) (30). This finding is closely aligned with the conclusions of our study. Our results indicate that, both before and after IPTW adjustment, patients receiving SRT+I exhibited superior overall survival (pre-IPTW: 26.7 months vs. 19.7 months, *P* = 0.012; post-IPTW: 29.3 months vs. 19.9 months, *P* = 0.034) and intracranial progression-free survival (pre-IPTW: 14.5 months vs. 8.4 months, *P* = 0.004; post-IPTW: 14.7 months vs. 9.4 months, *P* = 0.038) compared to those receiving WBRT+I in the overall cohort, an advantage that was maintained in the key subgroup with ≤4 brain metastases. Furthermore, the SRT+I group showed significantly better intracranial objective response rate (78.6% vs. 61.5%) and intracranial disease control rate (95.0% vs. 88.5%) compared to the WBRT+I group. These findings not only further validate the synergistic anti-tumor mechanisms of radiotherapy and immunotherapy but, more importantly, reveal the differential treatment effects produced by the combination of different radiotherapy techniques with immunotherapy. These results provide a solid evidence-based foundation for optimizing comprehensive treatment strategies for NSCLC patients with BMs and support the more precise selection of radiotherapy techniques in combination with I in clinical practice.

The temporal relationship between intracranial radiotherapy and I remains a subject of ongoing investigation in the management of BMs from NSCLC. Current evidence predominantly supports a concurrent treatment approach, typically characterized by an interval of ≤4 weeks between radiotherapy and I, which demonstrates significant advantages in OS, local control, and reduction of new BMs development (31, 32). However, within this concurrent treatment paradigm, the optimal timing window and administration sequence require further elucidation. Preclinical investigations indicate that CTLA-4 inhibitor administration following radiotherapy may achieve superior tumor growth suppression compared to pre-radiotherapy administration (33). Clinical observations parallel these findings. Scoccianti et al. reported that initiating I after (SRT→ICI) demonstrated a trend toward improved overall survival compared to the reverse sequence (HR = 0.74, P = 0.574) (34), with similar observations noted in patients receiving non-concurrent therapy (35). Our results align with these previous findings. We observed that patients initiating I within 3 months after completing their first intracranial radiotherapy course derived significantly greater survival benefits compared to those receiving I within 3 months before radiotherapy. The underlying mechanism for this sequential advantage may involve enhanced dendritic cell-mediated antigen cross-presentation and increased blood-brain barrier/blood-tumor barrier permeability following initial radiotherapy, thereby synergistically potentiating the anti-tumor activity of I (36).

In this study, immune-related adverse events (irAEs) in both treatment groups were primarily immune-mediated dermatitis and hypothyroidism, with the majority being mild in severity. Unfortunately, one patient in the WBRT+I group died due to grade ≥3 immune-mediated pneumonitis. Most notably, we observed a higher incidence of radiation-induced brain injury in the WBRT+I group compared to the SRT+I group (10.26% vs. 3.75%), suggesting a potential advantage for SRT+I in reducing radiation injury to brain tissue. This finding is consistent with previous studies demonstrating the superior cognitive preservation associated with stereotactic radiosurgery (37–39). These results indicate that the SRT+I regimen does not exacerbate radiotherapy-related toxicity, thereby further supporting the safety profile of this treatment strategy.

This study has several important limitations that should be considered when interpreting the results. First, the retrospective single-center design may introduce selection bias and unmeasured confounding factors. Although IPTW was applied for adjustment, residual confounding may persist. Furthermore, while we balanced the binary status of extracranial metastases via IPTW, our retrospective data extraction did not capture granular details regarding the specific organ sites of extracranial involvement (e.g., liver or bone), which could potentially influence systemic outcomes. Second, the moderate sample size may reduce statistical power and limit the robustness of the conclusions, indicating the need for validation through larger multicenter prospective studies. Regarding the study design, while our strict inclusion criteria—restricting to patients receiving their first intracranial radiotherapy with I within 3 months before or after radiotherapy—facilitated the evaluation of efficacy in a specific treatment context, this approach did not adequately assess the potential impacts of systemic therapies such as anti-angiogenic treatments or subsequent salvage radiotherapy on survival outcomes. Additionally, retrospective data collection relying on a single medical center’s electronic medical record system may have resulted in incomplete documentation of immunotherapy-related adverse events, potentially leading to an underestimation of their actual incidence. Moreover, standardized neurocognitive assessments and formal patient-reported outcomes (PROs) were not routinely documented in the retrospective records. Consequently, our safety endpoints rely primarily on documented radiological and severe clinical toxicities, preventing a comprehensive evaluation of the cognitive preservation benefits typically associated with SRT. Finally, with approximately two-thirds of the study population being under 65 years of age, this age distribution may limit the generalizability of the findings to real-world NSCLC populations. Despite these limitations, the core findings of this study still provide valuable evidence for clinical practice.

In conclusion, this systematic analysis demonstrates that SRT+I shows significant advantages over WBRT+I in patients with driver gene-negative NSCLC with BMs. In the overall cohort analysis, the SRT+I group achieved significantly superior median OS and iPFS compared to the WBRT+I group, with this advantage remaining consistent in the key subgroup of patients with ≤4 BMs. In the I sequence subgroup analysis, administering I following initial intracranial radiotherapy demonstrated particularly notable survival benefits. Regarding tumor response, the SRT+I group achieved higher iORR and iDCR, along with a significantly reduced risk of intracranial disease progression. Safety analysis revealed a significantly lower incidence of documented radiation-induced brain injury in the SRT+I group compared to the WBRT+I group, with no grade ≥3 irAEs reported, indicating a favorable safety profile within the limits of our retrospective evaluation. These results robustly demonstrate that the SRT+I combination regimen provides clear survival benefits and acceptable safety in patients with driver gene-negative NSCLC with BMs, offering valuable evidence for clinical decision-making.

## Data Availability

The raw data supporting the conclusions of this article will be made available by the authors, without undue reservation.

## Supplementary material

Supplementary Figure 1 Variable selection using the LASSO regression model in the overall cohort, showing the coefficient profiles and cross-validation process.

Supplementary Figure 2 Variable selection using the LASSO regression model in the subgroup with ≤4 brain metastases, showing the coefficient profiles and cross-validation process.

Supplementary Figure 3 Multivariable Cox regression analysis of prognostic factors for OS and iPFS before and after IPTW adjustment in the subgroup with ≤4 BMs. **(A)** OS before IPTW. **(B)** OS after IPTW. **(C)** iPFS before IPTW. **(D)** iPFS after IPTW.

Supplementary Figure 4 Variable selection using the LASSO regression model in the I sequence cohort, showing the coefficient profiles and cross-validation process.

Supplementary Figure 5 Multivariable Cox regression analysis of prognostic factors for OS and iPFS before and after IPTW adjustment in the I sequence cohort. **(A)** OS before IPTW. **(B)** OS after IPTW. **(C)** iPFS before IPTW. **(D)** iPFS after IPTW.

Supplementary Table 1 In the subgroup with ≤4 BMs, patients’ demographics and baseline characteristics before and after IPTW.

Supplementary Table 2 In the I sequence subgroup, patients’ demographics and baseline characteristics before and after IPTW.

Supplementary Table 3 Intracranial tumor response.
