# Supplementary figures and images for: Stereotactic versus whole-brain radiotherapy combined with immunotherapy in driver gene–negative NSCLC with brain metastases: a real-world IPTW analysis
Source: Front Immunol. 2026 Jun 22;17:1815565. doi: 10.3389/fimmu.2026.1815565 (PMC13333633; doi:10.3389/fimmu.2026.1815565)

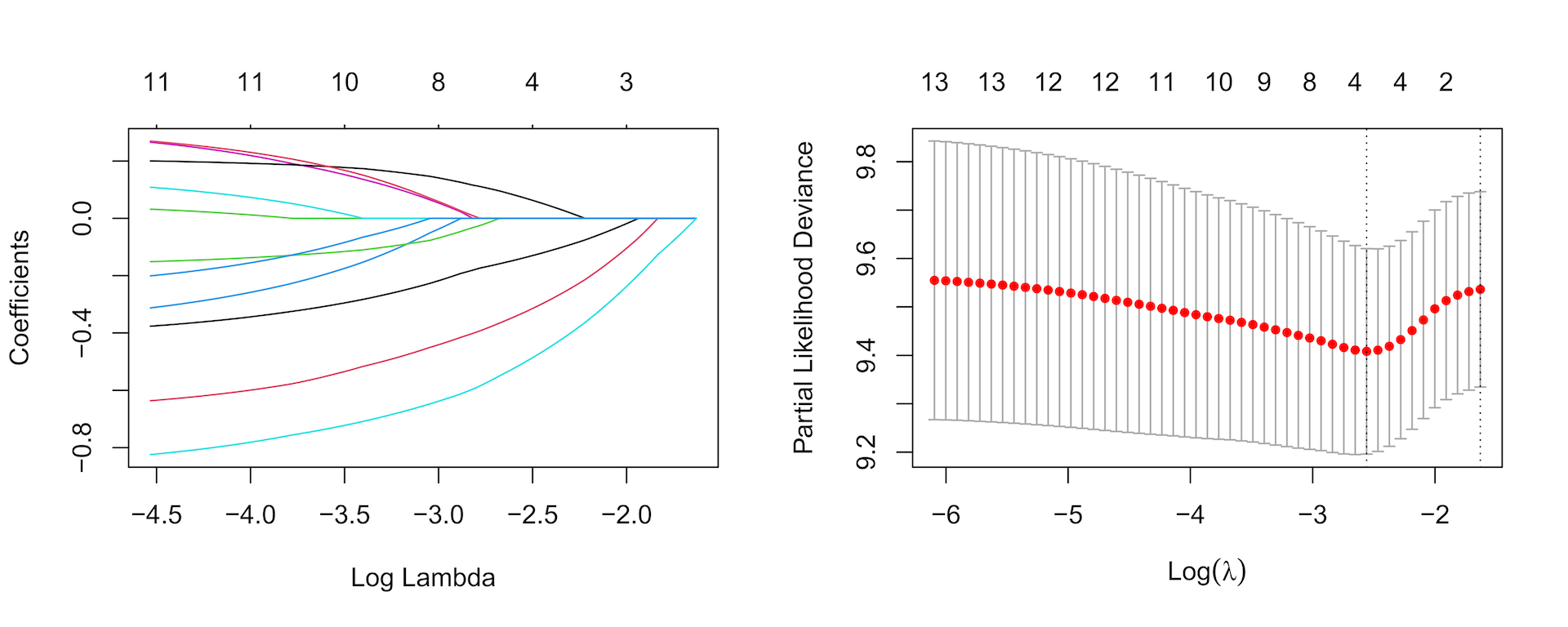

Supplement: Supplementary Figure 1 — Variable selection using the LASSO regression model in the overall cohort, showing the coefficient profiles and cross-validation process. [file Image1.tif]

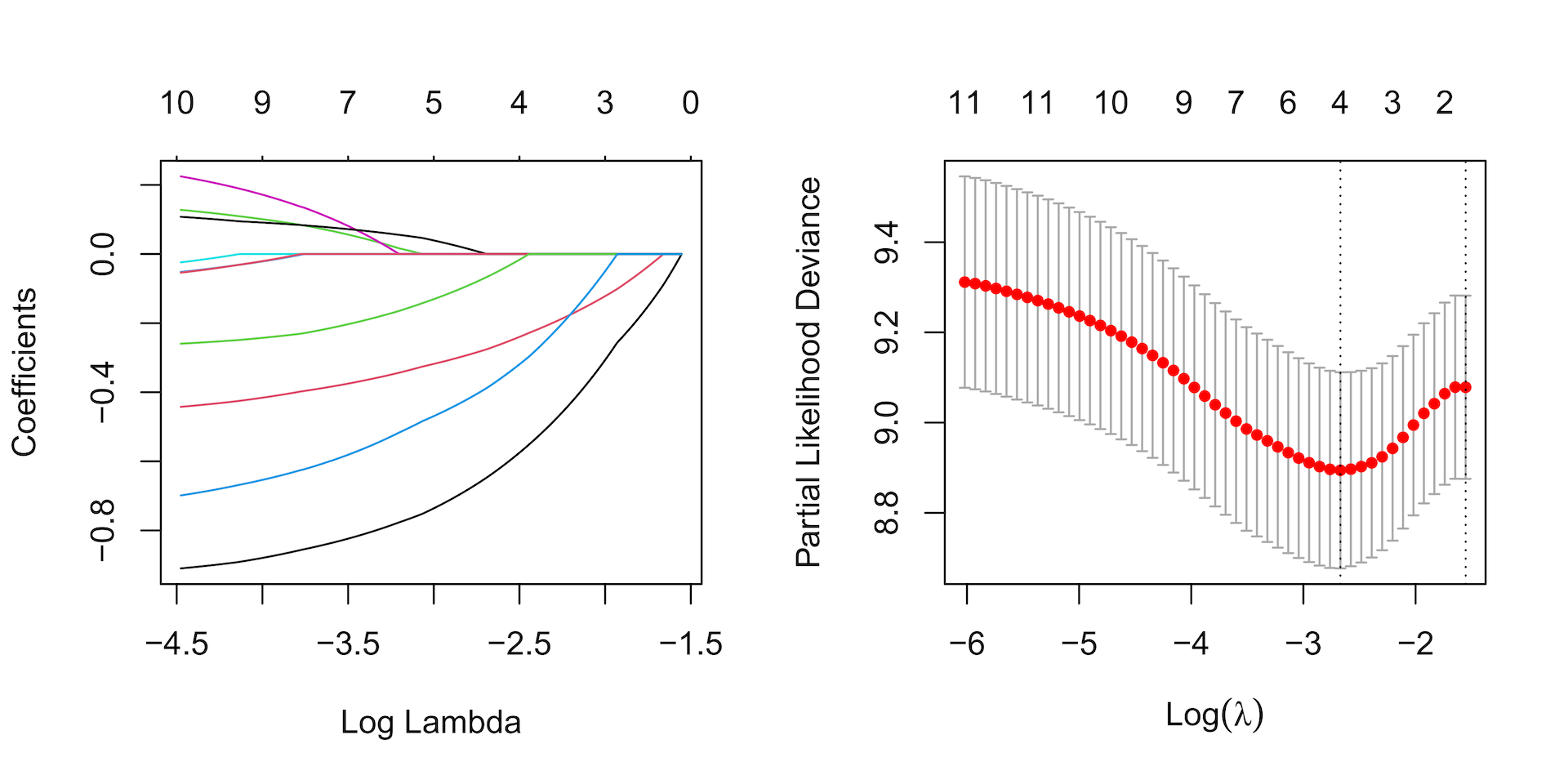

Supplement: Supplementary Figure 2 — Variable selection using the LASSO regression model in the subgroup with ≤4 brain metastases, showing the coefficient profiles and cross-validation process. [file Image2.tif]

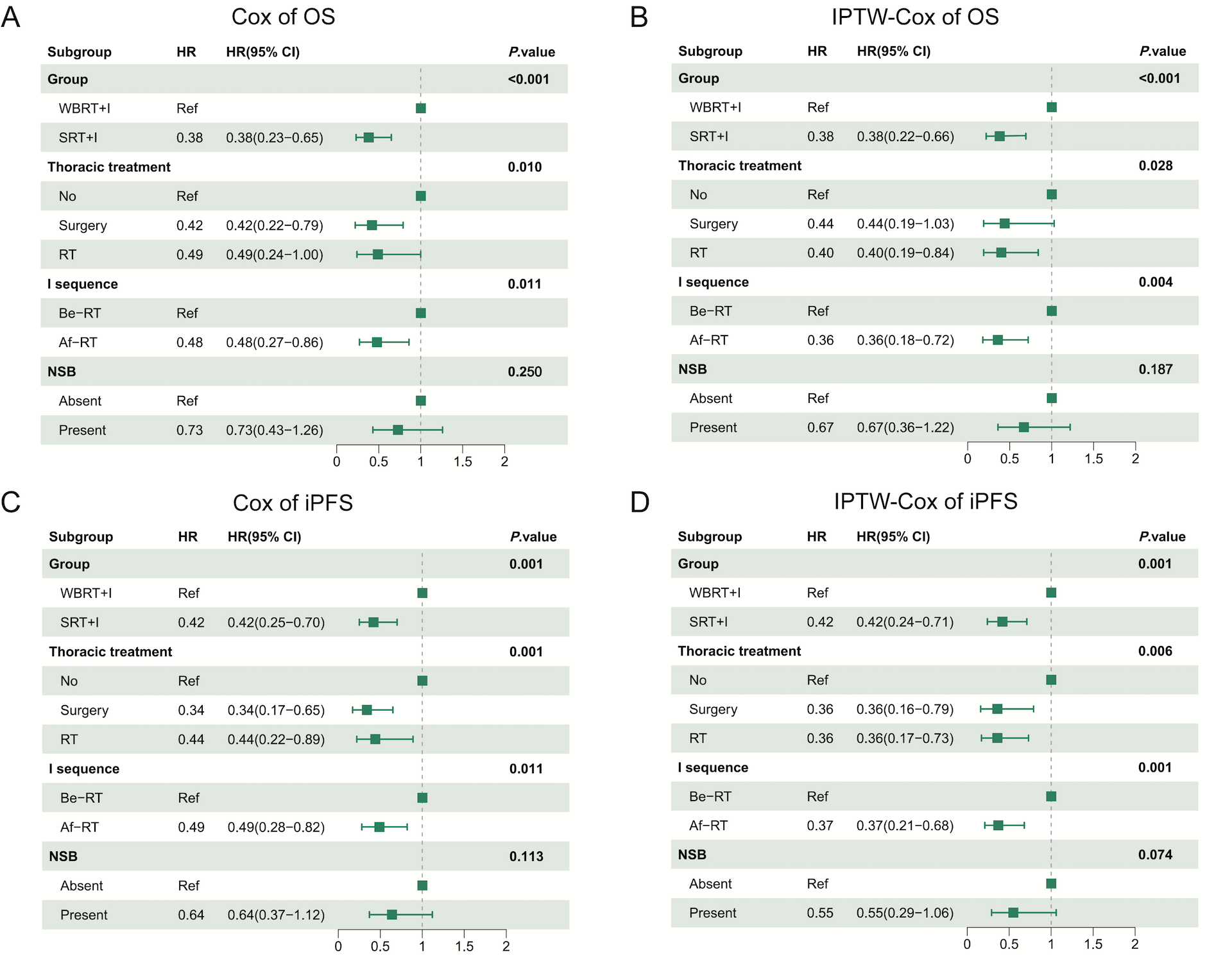

Supplement: Supplementary Figure 3 — Multivariable Cox regression analysis of prognostic factors for OS and iPFS before and after IPTW adjustment in the subgroup with ≤4 BMs. (A) OS before IPTW. (B) OS after IPTW. (C) iPFS before IPTW. (D) iPFS after IPTW. [file Image3.tif]

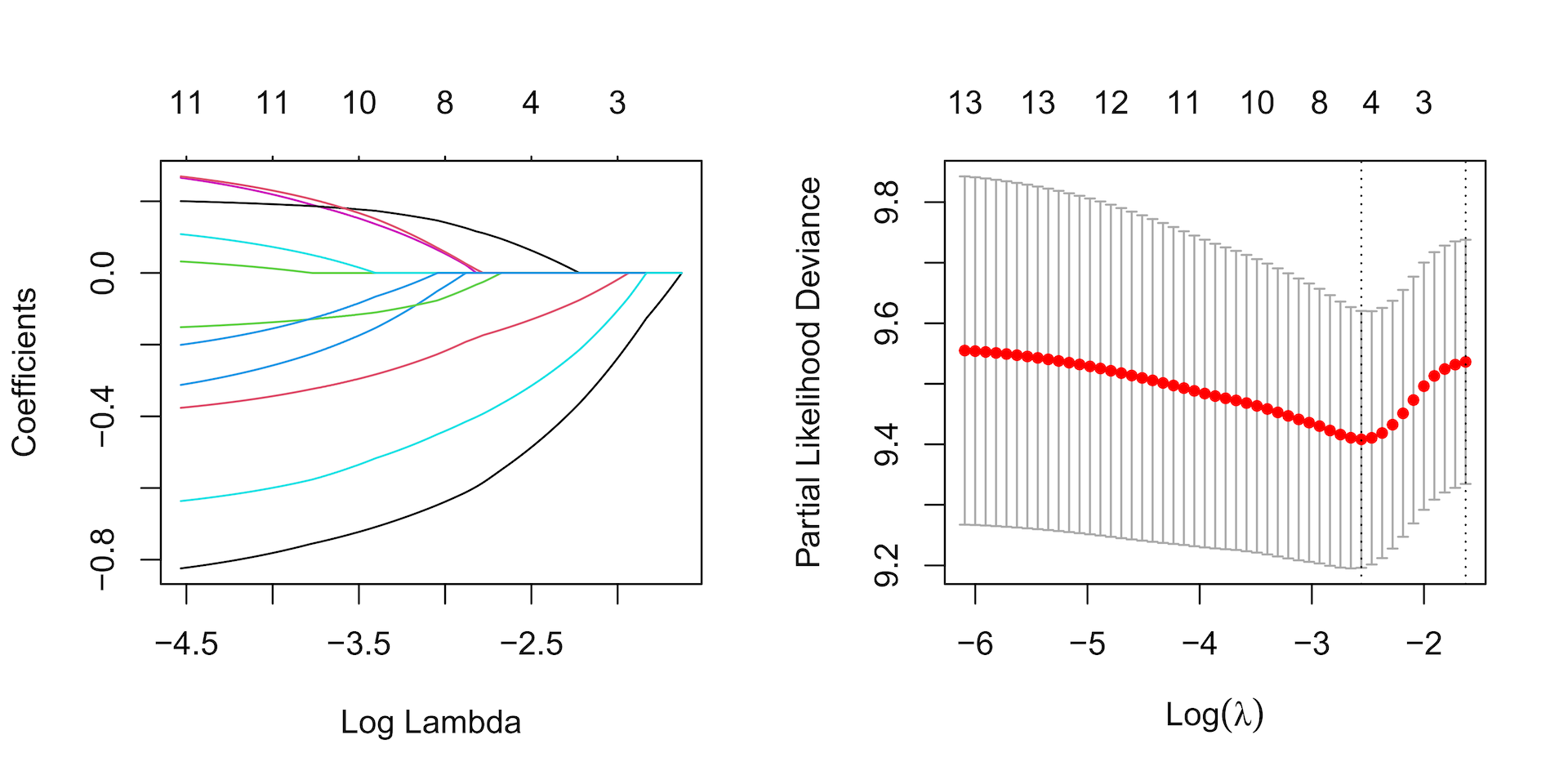

Supplement: Supplementary Figure 4 — Variable selection using the LASSO regression model in the I sequence cohort, showing the coefficient profiles and cross-validation process. [file Image4.tif]

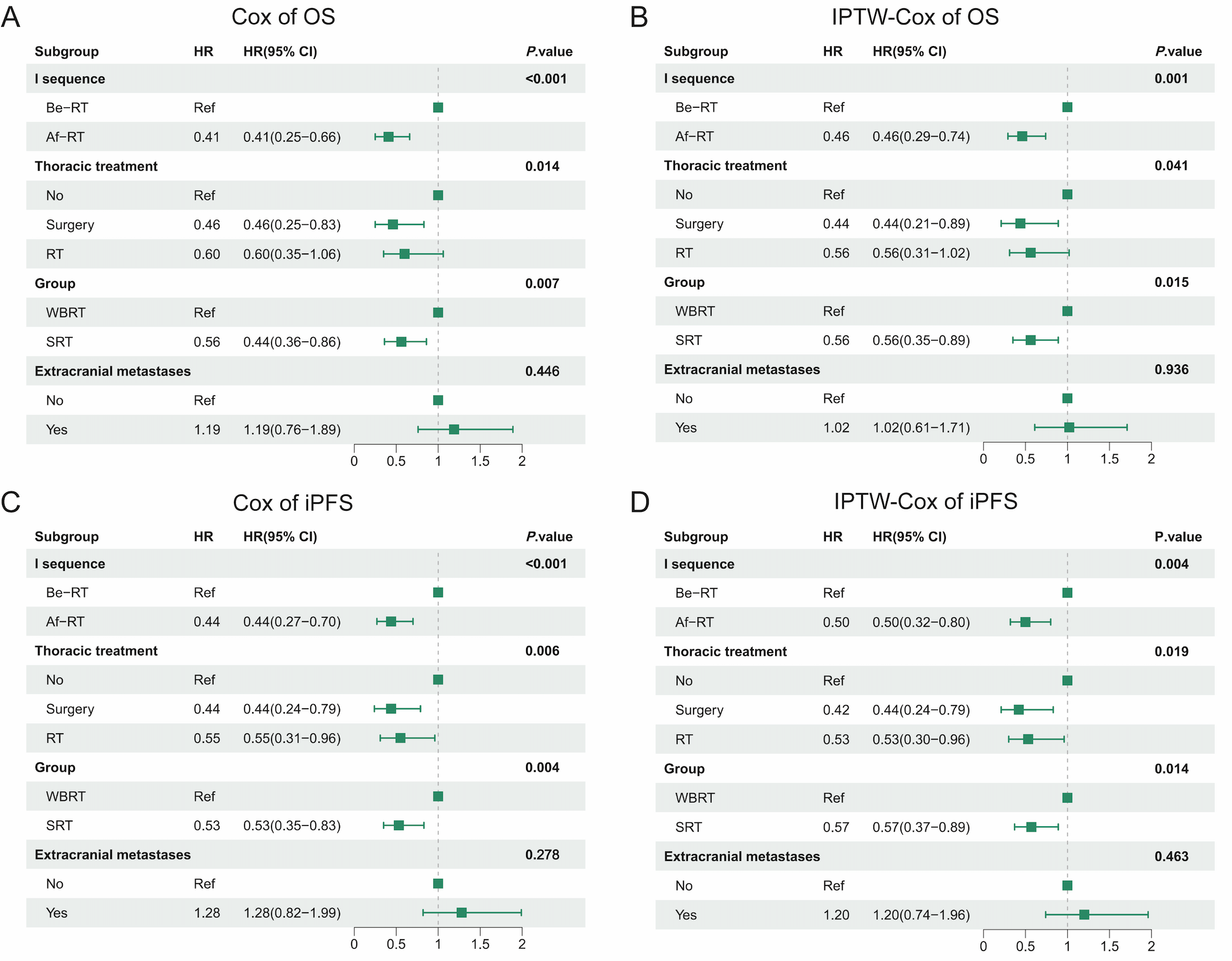

Supplement: Supplementary Figure 5 — Multivariable Cox regression analysis of prognostic factors for OS and iPFS before and after IPTW adjustment in the I sequence cohort. (A) OS before IPTW. (B) OS after IPTW. (C) iPFS before IPTW. (D) iPFS after IPTW. [file Image5.tif]
