# Supplementary material for: Stereotactic versus whole-brain radiotherapy combined with immunotherapy in driver gene–negative NSCLC with brain metastases: a real-world IPTW analysis
Source: Front Immunol. 2026 Jun 22;17:1815565. doi: 10.3389/fimmu.2026.1815565 (PMC13333633; doi:10.3389/fimmu.2026.1815565)
Supplement: Supplementary Table 1 — In the subgroup with ≤4 BMs, patients’ demographics and baseline characteristics before and after IPTW. [file Table1.docx]

| **Characteristic** | **Before IPTW** | | ***P*-value** | **SMD** | **After IPTW** | | ***P*-value** | **SMD** |
| --- | --- | --- | --- | --- | --- | --- | --- | --- |
|  | **WBRT+I, N = 40** | **SRT+I, N = 72** |  |  | **WBRT+I, N = 102.6** | **SRT+I, N = 113.1** |  |  |
| **Sex** |  |  | 0.043 | 0.438 |  |  | 0.967 | 0.010 |
| **Male** | 28 (70.0%) | 63 (87.5%) |  |  | 82.1 (80.0%) | 90.9 (80.4%) |  |  |
| **Female** | 12 (30.0%) | 9 (12.5%) |  |  | 20.5 (20.0%) | 22.2 (19.6%) |  |  |
| **Age** |  |  | 0.025 | 0.508 |  |  | 0.683 | 0.101 |
| **<65** | 31 (77.5%) | 39 (54.2%) |  |  | 70.4 (68.6) | 72.1 (63.8) |  |  |
| **≥65** | 9 (22.5) | 33 (45.8) |  |  | 32.2 (31.4) | 41.0 (36.2) |  |  |
| **KPS** |  |  | 0.282 | 0.254 |  |  | 0.715 | 0.088 |
| **<80** | 15 (37.5%) | 36 (50.0%) |  |  | 44.8 (43.7%) | 54.3 (48.1%) |  |  |
| **≥80** | 25 (62.5%) | 36 (50.0%) |  |  | 57.8 (56.3%) | 58.8 (51.9%) |  |  |
| **Pathology** |  |  | 0.655 | 0.134 |  |  | 0.973 | 0.007 |
| **Adenocarcinoma** | 30 (75.0%) | 58 (70.6%) |  |  | 83.1 (81.0%) | 91.3 (80.7%) |  |  |
| **Squamous** | 10 (25.0%) | 14 (19.4%) |  |  | 19.5 (19.0%) | 21.8 (19.3%) |  |  |
| **Extracranial metastases** |  |  | 0.189 | 0.304 |  |  | 0.417 | 0.081 |
| **No** | 13 (32.5%) | 36 (47.2%) |  |  | 31.8 (31.0%) | 44.8 (39.6%) |  |  |
| **Yes** | 27 (67.5%) | 38 (52.8%) |  |  | 70.8 (69.0%) | 68.3 (60.4%) |  |  |
| **Edema of BMs** |  |  | 0.607 | 0.152 |  |  | 0.933 | 0.020 |
| **Absent** | 7 (17.5%) | 17 (23.6%) |  |  | 20.5 (20.0%) | 23.5 (20.8%) |  |  |
| **Present** | 33 (82.5%) | 55 (76.4%) |  |  | 82.1 (80.0%) | 89.6 (79.2%) |  |  |
| **BMs type** |  |  | 0.844 | 0.070 |  |  | 0.701 | 0.087 |
| **Synchronous** | 19 (47.5%) | 37 (51.4%) |  |  | 59.9 (58.4) | 61.1 (54.0) |  |  |
| **Metachronous** | 21 (52.5%) | 35 (48.6%) |  |  | 42.7 (41.6) | 52.0 (46.0) |  |  |
| **D-max** |  |  | 0.668 | 0.124 |  |  | 0.721 | 0.084 |
| **<1.8cm** | 22 (55.0%) | 46 (61.1%) |  |  | 56.7 (55.2%) | 67.2 (59.4%) |  |  |
| **≥1.8cm** | 18 (45.0%) | 28 (38.9%) |  |  | 45.9 (44.8%) | 45.9 (40.6%) |  |  |
| **Thoracic treatment** |  |  | 0.066 | 0.478 |  |  | 0.807 | 0.051 |
| **No** | 28 (70.0%) | 34 (47.2%) |  |  | 63.6 (62.0%) | 63.3 (56.0%) |  |  |
| **Surgery** | 8 (20.0%) | 24 (33.3%) |  |  | 27.7 (27.0%) | 32.1 (28.3%) |  |  |
| **RT** | 4 (10.0%) | 14 (19.4%) |  |  | 11.3 (11.0%) | 17.7 (15.7%) |  |  |
| **NSB** |  |  | 0.223 | 0.281 |  |  | 0.611 | 0.118 |
| **Absent** | 23 (57.5%) | 51 (70.8%) |  |  | 68.4 (66.7%) | 81.6 (72.1%) |  |  |
| **Present** | 17 (42.5%) | 21 (29.2%) |  |  | 34.2 (33.3%) | 31.5 (27.9%) |  |  |
| **PD(L)-1(TPS)** |  |  | 0.722 | 0.162 |  |  | 0.969 | 0.053 |
| **<1.0%** | 6 (15.0%) | 15 (20.8%) |  |  | 18.5 (18.0%) | 21.3 (18.8%) |  |  |
| **≥1.0%** | 8 (20.0%) | 12 (16.7%) |  |  | 14.9 (14.5%) | 18.1 (16.1%) |  |  |
| **Untested** | 26 (65.0%) | 45 (62.5%) |  |  | 69.2 (67.5%) | 73.7 (65.1%) |  |  |
| **I sequence** |  |  | 0.139 | 0.332 |  |  | 0.733 | 0.083 |
| **Be-RT** | 22(55.0%) | 51 (70.8%) |  |  | 60.3 (58.8%) | 71.1 (62.9%) |  |  |
| **Af-RT** | 18 (45.0%) | 21 (29.2% |  |  | 42.3 (41.2%) | 42.0 (37.1%) |  |  |
