# Supplementary material for: Stereotactic versus whole-brain radiotherapy combined with immunotherapy in driver gene–negative NSCLC with brain metastases: a real-world IPTW analysis
Source: Front Immunol. 2026 Jun 22;17:1815565. doi: 10.3389/fimmu.2026.1815565 (PMC13333633; doi:10.3389/fimmu.2026.1815565)
Supplement: Supplementary Table 2 — In the I sequence subgroup, patients’ demographics and baseline characteristics before and after IPTW. [file Table2.docx]

| **Characteristic** | **Before IPTW** | | ***P*-value** | **SMD** | **After IPTW** | | ***P*-value** | **SMD** |
| --- | --- | --- | --- | --- | --- | --- | --- | --- |
|  | **Be-RT, N = 104** | **Af-RT, N = 54** |  |  | **Be-RT, N = 155.9** | **Af-RT, N = 159.5** |  |  |
| **Sex** |  |  | 0.198 | 0.247 |  |  | 0.939 | 0.013 |
| **Male** | 89 (85.6%) | 41 (75.9%) |  |  | 129.9 (83.3%) | 132.1 (82.8%) |  |  |
| **Female** | 15 (14.4%) | 13 (24.1%) |  |  | 26.0 (16.7%) | 27.4 (17.2%) |  |  |
| **Age** |  |  | 0.732 | 0.087 |  |  | 0.985 | 0.003 |
| **<65** | 64 (62.5%) | 36 (67.4%) |  |  | 98.5 (63.2%) | 100.5 (63.0%) |  |  |
| **≥65** | 39 (37.5%) | 18 (33.3%) |  |  | 57.4 (36.8%) | 59.0 (37.0%) |  |  |
| **KPS** |  |  | 0.778 | 0.076 |  |  | 0.550 | 0.011 |
| **<80** | 49 (48.1%) | 28 (49.9%) |  |  | 77.8 (49.9%) | 88.4 (55.4%) |  |  |
| **≥80** | 54 (51.9)% | 26 (48.1%) |  |  | 78.1 (50.1%) | 71.1 (44.6%) |  |  |
| **Pathology** |  |  | 0.748 | 0.088 |  |  | 0.873 | 0.030 |
| **Adenocarcinoma** | 79 (76.0%) | 43 (79.6%) |  |  | 121.0 (77.6%) | 125.8 (78.9%) |  |  |
| **Squamous** | 25 (24.0%) | 11 (20.4%) |  |  | 34.9 (22.4%) | 33.7 (21.1%) |  |  |
| **Extracranial metastases** |  |  | 0.633 | 0.109 |  |  | 0.908 | 0.021 |
| **No** | 34 (33.7%) | 21 (38.9%) |  |  | 54.1 (34.7%) | 53.8 (33.7%) |  |  |
| **Yes** | 69 (66.3%) | 33 (61.1%) |  |  | 101.8 (65.3%) | 105.7 (66.3%) |  |  |
| **BMs count** |  |  | 0.412 | 0.171 |  |  | 0.933 | 0.016 |
| **≤4** | 71 (69.3%) | 41 (75.9%) |  |  | 109.7 (70.3%) | 111.0 (69.6%) |  |  |
| **>4** | 33 (31.7%) | 13 (24.1%) |  |  | 46.2 (29.7%) | 48.5 (30.4%) |  |  |
| **Edema of BMs** |  |  | 0.961 | 0.043 |  |  | 0.797 | 0.047 |
| **Absent** | 22 (22.1%) | 11 (21.4%) |  |  | 32.8 (21.0%) | 30.5 (19.2%) |  |  |
| **Present** | 81 (77.9%) | 43 (79.6%) |  |  | 123.1 (79.0%) | 129.0 (80.8%) |  |  |
| **BMs type** |  |  | 0.065 | 0.343 |  |  | 0.968 | 0.007 |
| **Synchronous** | 45 (54.2%) | 33 (61.1%) |  |  | 76.8 (49.3%) | 78.0 (48.9%) |  |  |
| **Metachronous** | 58 (55.8%) | 21 (38.9%) |  |  | 79.1 (50.7%) | 81.5 (51.1%) |  |  |
| **D-max** |  |  | 0.436 | 0.159 |  |  | 0.755 | 0.056 |
| **<1.8cm** | 63 (61.5%) | 29 (43.7%) |  |  | 93.6 (60.0%) | 100.1 (62.8%) |  |  |
| **≥1.8cm** | 40 (38.5%) | 25 (46.3%) |  |  | 62.3 (40.0%) | 59.4 (37.2%) |  |  |
| **Thoracic treatment** |  |  | 0.824 | 0.105 |  |  | 0.974 | 0.042 |
| **No** | 63 (60.6%) | 33 (61.1%) |  |  | 93.7 (60.1%) | 98.5 (61.7%) |  |  |
| **Surgery** | 22 (21.2%) | 13 (24.1%) |  |  | 34.1 (22.1%) | 32.6 (20.4%) |  |  |
| **RT** | 19 (18.3%) | 8 (14.8%) |  |  | 27.8 (17.8%) | 28.4 (17.8%) |  |  |
| **NSB** |  |  | 0.109 | 0.298 |  |  | 0.971 | 0.007 |
| **Absent** | 68 (66.3%) | 28 (51.9%) |  |  | 97.6 (62.6%) | 99.3 (62.3%) |  |  |
| **Present** | 35 (33.7%) | 26 (48.1%) |  |  | 58.3 (37.4%) | 60.2 (37.7%) |  |  |
| **PD(L)-1(TPS)** |  |  | 0.212 | 0.313 |  |  | 0.966 | 0.048 |
| **<1.0%** | 21 (20.2%) | 5 ( 9.3%) |  |  | 26.0 (16.7%) | 24.9 (15.6%) |  |  |
| **≥1.0%** | 21 (20.2%) | 12 (22.2%) |  |  | 32.1 (20.6%) | 30.9 (19.4%) |  |  |
| **Untested** | 62 (59.6%) | 37 (68.5%) |  |  | 97.8 (62.7%) | 103.7 (65.1%) |  |  |
| **Group** |  |  | 0.197 | 0.246 |  |  | 0.775 | 0.053 |
| **WBRT+I** | 46 (55.0%) | 31 (47.4%) |  |  | 74.9 (48.0%) | 80.9 (50.7%) |  |  |
| **SRT+I** | 57 (54.8%) | 23 (42.6%) |  |  | 81.0 (52.0%) | 78.6 (49.3%) |  |  |
