# Supplementary material for: Stereotactic versus whole-brain radiotherapy combined with immunotherapy in driver gene–negative NSCLC with brain metastases: a real-world IPTW analysis
Source: Front Immunol. 2026 Jun 22;17:1815565. doi: 10.3389/fimmu.2026.1815565 (PMC13333633; doi:10.3389/fimmu.2026.1815565)
Supplement: Supplementary Table 3 — Intracranial tumor response. [file Table3.docx]

| **Outcomes** | **WBRT+I (N=78)** | **SRT+I (N=80)** |
| --- | --- | --- |
| Best of intracranial response |  |  |
| CR | 2 (2.6%) | 7 (8.8%) |
| PR | 46 (59.0%) | 56 (70.0%) |
| SD | 21 (26.9%) | 13 (16.2%) |
| PD | 9 (11.5%) | 4 (5.0%) |
| iORR, n (%),  95% CI | 48 (61.5)  49.8-72.3 | 63(78.6)  68.2-87.1 |
| iDCR, n (%),  95% CI | 69 (88.5)  79.2-94.6 | 76 (95.0)  87.7-98.6 |
| iPDR, n (%),  95% CI | 9 (11.5)  5.4-20.8 | 4 (5.0)  1.4-12.3 |

CI, confidence interval; CR, complete response; PD, progressive disease; PR, partial response; SD, stable disease; iORR, intracranial objective response rate; iDCR, intracranial disease control rate; iDPR, intracranial disease progression rate.
